# Codes, Clues, and Checkups: Teaching Pediatric Health Supervision to Medical Students with an Escape Room

**DOI:** 10.15766/mep_2374-8265.11653

**Published:** 2026-11-13

**Authors:** Michelle Shiffman, Jennifer Soep, Tai Lockspeiser, Anne Frank

**Affiliations:** 1 Assistant Professor, Department of Pediatrics, Denver Health; University of Colorado; 2 Professor, Department of Pediatrics, Children's Hospital Colorado; University of Colorado; 3 Professor, Department of Pediatrics, Children's Hospital Colorado; University of Colorado; 4 Associate Professor, Department of Medicine and Pediatrics, Denver Health; University of Colorado

**Keywords:** Escape Room, Developmental Milestones, Pediatric Anticipatory Guidance, Games, Vaccine Hesitancy, HEEADSSS Psychosocial Screening, Pediatric Nutrition

## Abstract

**Introduction:**

Pediatric health supervision, including developmental surveillance, preventive care, and anticipatory guidance, is essential to pediatric practice. However, it can be difficult to teach medical students in a consistent and clinically relevant way, particularly when their exposure to well-child care varies by site.

**Methods:**

We developed a 2-hour pediatric health supervision escape room for medical students during their clerkship year. Students completed presession materials and progressed through 5 age-based “rooms.” Activities required use of clinical resources and addressed developmental surveillance, anticipatory guidance, nutrition, oral health, injury prevention, vaccine counseling, and psychosocial screening. The activity concluded with a facilitator-led debrief. Postsession evaluations included Likert-style questions addressing Kirkpatrick Level 1 (reaction) and optional open-ended questions capturing self-reported takeaways. Facilitator feedback was sought via open-ended responses.

**Results:**

Over one academic year, 168 students participated, and 142 completed postsession evaluations (84.5%). Reaction outcomes were strong: 86.6% rated overall session quality as very good or excellent, 81.7% agreed the session improved preparedness for preventive care visits, 88.7% agreed it helped them learn clinically relevant resources, and 93.7% agreed it helped them identify knowledge gaps. Open-ended responses provided illustrative examples of self-reported takeaways aligned with the learning objectives. Learners also emphasized the value of the interactive, team-based format. Faculty reported high engagement and enjoyment of the session.

**Discussion:**

This escape room provides an engaging, low-technology approach to teaching pediatric health supervision. Students valued the active format and reported clinically relevant takeaways for future practice. This model may be adaptable across institutions and learner levels.

## Educational Objectives

By the end of this activity, learners will be able to:

1. Identify normal developmental milestones across gross motor, fine motor, verbal, and social domains for a given age, using a standardized surveillance tool (eg, CDC Milestone Moments).
2. Select the components of a well-child visit appropriate for a given age, including anticipatory guidance for nutrition, oral health, and safety, using the AAP Bright Futures guidelines and Periodicity Schedule.
3. Name the unique components of an adolescent well-child check, including the HEEADSSS psychosocial interview (Home, Education/Employment, Eating, Activities, Drugs, Sexuality, Suicide/Depression, and Safety).
4. Describe the recommended childhood vaccine schedule, and apply presumptive and motivational interviewing language to address a common vaccine-related question.

## Introduction

Pediatric health supervision visits are complex. Clinicians must integrate evidence-based guidance with growth monitoring, developmental surveillance, and family dynamics while delivering age-appropriate anticipatory and safety guidance. The American Academy of Pediatrics (AAP) outlines comprehensive recommendations for preventive pediatric care in the Bright Futures guidelines and Periodicity Schedule.^1-3^ Yet survey and focus-group studies show that families often do not receive complete or consistent anticipatory guidance from their pediatrician,^4-6^ suggesting that preventive care takes more than familiarity with the guidelines.

Despite their central role in pediatric care, these topics are frequently underemphasized in undergraduate medical education. Students frequently report feeling underprepared for preventive care visits and lack confidence in applying pediatric guidelines clinically.^7-9^ The Council on Medical Student Education in Pediatrics (COMSEP) includes components of these guidelines as essential competencies for clerkship-year students.^10^ Implementing these recommendations requires deliberate strategies that emphasize applying knowledge clinically.

Prior educational approaches to pediatric health supervision have primarily used lecture-based or case-based formats.^11,12^ Some curricula have focused on individual domains such as developmental milestones, vaccine counseling, or the HEEADSSS psychosocial interview (Home, Education/Employment, Eating, Activities, Drugs, Sexuality, Suicide/Depression, and Safety), but these are often taught in isolation.^13-15^ These approaches do not always address the complexity of pediatric health supervision or the need to integrate guidelines across patients. Many curricula also rely on passive rather than active, applied learning, which may limit learners’ ability to apply these concepts clinically. Given the wide variation in patient mix, preceptor style, and exposure to well-child visits across training sites, an engaging, accessible curriculum is especially valuable.

Game-based learning, including the use of escape rooms, is occurring more often in health care professional curricula.^16-19^ Escape rooms have been used to teach professional students many topics, including basic concepts in infectious disease,^20^ head and neck anatomy,^21^ patient safety,^22^ and critical thinking in emergency medicine.^23^ Game-based learning and escape rooms incorporate elements of self-directed learning theory by allowing adult learners to be autonomous, identify learning gaps, and work through problems relevant to professional practice.^24^ Games and escape rooms also offer opportunities to utilize intrinsic motivation, competence, mastery, and immediate feedback.^25^ Building on these principles, we designed an escape room–based educational session focused on pediatric health supervision.

## Methods

### Activity Design and Development

We, the author team of pediatric clerkship faculty, along with help from a dedicated resident and medical student (see Acknowledgments), designed this activity using Kern's six-step model^26^ to strengthen how pediatric health supervision is taught in our clerkship curriculum.

### Curricular Context and Learner Preparation

Second-year medical students at the University of Colorado completed their core clerkship year across multiple clinical sites throughout Colorado. As part of a 44-week didactic curriculum spanning Pediatrics, Psychiatry, Family Medicine, Surgery, Emergency Medicine, Obstetrics and Gynecology, and Internal Medicine, we developed a 2-hour, 5-station escape room to teach pediatric health supervision using small-group competition followed by a facilitator-led debrief.

Students had received foundational instruction on vaccines, puberty, nutrition, and psychosocial screening during preclinical coursework, though outpatient exposure varied at the start of the session. Students could optionally complete approximately 30 to 90 minutes of preparatory work beforehand, depending on how much they chose to review: a breastfeeding slide deck (Appendix A), and open-access reference articles on oral health, developmental milestones, nutrition guidance, safe sleep, safe firearm storage, and child passenger safety,^27-31^ provided to students as direct links.

Facilitators were pediatric faculty who reviewed session materials and slides in advance, although no formal facilitator training was required. Facilitators reported that reviewing session materials took about 10 minutes. Clerkship coordinators assembled the materials for each age-based room (baby, toddler, kindergarten, fourth grade, and eighth grade) and a shared toolkit, using a standardized guide (Appendix B); it was thought to take roughly 30 minutes to print and assemble the materials for a group of 30 students, and the only cost was for printing materials.

As this study involved analyses of deidentified educational data, it was deemed exempt by the Colorado Multiple Institution Review Board (#23-1664).

### Implementation

Facilitators reviewed the escape room guidelines with students at the start of the session (Appendix C) and instructed them to work collaboratively, without external resources. Students were divided into groups of 3 to 6, each stationed in the shared activity space and given the materials for the first room (eg, the baby room). A team had to complete all puzzles in a room before a facilitator checked their answers and released the materials for the next room. Sessions typically ran with 4 to 5 groups competing simultaneously, supervised by 1 to 2 facilitators.

The narrative premise was that extraterrestrial beings had invaded the neighborhood day care and schools. Teams completed challenges in each room to “rescue” the children. We chose this narrative rather than a realistic clinic scenario to lower the stakes of an incorrect answer during a timed, competitive activity. Programs preferring a clinically grounded frame could adapt the same puzzles around a clinic-visit narrative instead.

Each team progressed through the 5 age-based rooms with 2 to 4 puzzles per room (see Appendix D for a description of each room's puzzles). Puzzles had to be solved correctly before a facilitator would mark them done and let the team move on; therefore, puzzle completion served as the initial, team-level indicator that an objective had been addressed. We did not separately confirm that every team member could perform each skill independently (see Limitations). Facilitators could verify answers using the answer key (Appendix D) and offer hints at their discretion if a team struggled with a puzzle. Hint guidance was not standardized during the evaluated cohort; however, examples have now been included in Appendix D (see Limitations). The first team to complete all rooms correctly was declared the winner based solely on order of completion, and no point-based scoring system was used.

Two hours were allotted for the entire activity, including debrief. A midpoint time check identified teams needing additional support. Based on facilitator reports, approximately 80%-90% of teams finished within the allotted time, though this was not formally tracked. After all teams finished, facilitators led a debrief using the same age-based content-review slides provided for facilitator preparation (Appendix D, slides 85-107), covering key clinical concepts from each room. Facilitators were told to reserve time for the debrief and review materials in advance, but had no standardized script to follow, so what they covered was left to their discretion. To standardize this component going forward, we developed a facilitator guide with structured debrief content (Appendix E) that organizes this same slide content into a consistent facilitator script. This guide has not yet been piloted and is presented as a proposed next step rather than a description of the debrief as delivered during the evaluated cohort. We used a generative AI tool (Claude Opus 5.5, Anthropic) to help organize our existing slide content into this guide; the author team reviewed, revised, and verified all content.

We piloted this escape room with 2 cohorts of students. The first year, we had no formal questionnaire, so only data from the second cohort, for which we had a structured feedback form, are included in this article. We also informally piloted the curriculum after the first year with 8 medicine/pediatrics residents, who gave feedback through a general open-text form; this feedback informed changes to our content for the described cohort, particularly the developmental milestones activity.

### Assessment and Evaluation

Students completed a postsession evaluation created by the author team with Likert-style questions addressing Kirkpatrick Level 1 (reaction) and optional open-ended questions capturing self-reported takeaways, aligned with the 4 learning objectives above (Appendix F). Because the open-ended items were optional, response rates were lower than the Likert-style items. Therefore, we chose to report sample quotes rather than response frequencies (see Results). Likert-style responses were analyzed with descriptive statistics. Open-ended responses from the students and facilitators were coded deductively based on which of the learning objectives the student mentioned, and the author team identified representative quotes.

## Results

Over one academic year, 168 second-year medical students participated in the Pediatric Health Supervision Escape Room. Postsession evaluations were completed by 142 of the students, for a response rate of 84.5%.

### Learner Reaction

At the level of Kirkpatrick Level 1 (reaction), learner responses were strongly positive (Table 1). Overall, 123 of 142 respondents (87%) rated session quality as very good or excellent. Most learners also agreed or strongly agreed that the session prepared them for preventive care visits (116/142, 82%), helped them learn about clinical resources relevant to pediatric health supervision (126/142, 89%), and helped them identify gaps in their knowledge (133/142, 94%).

**Table 1.**
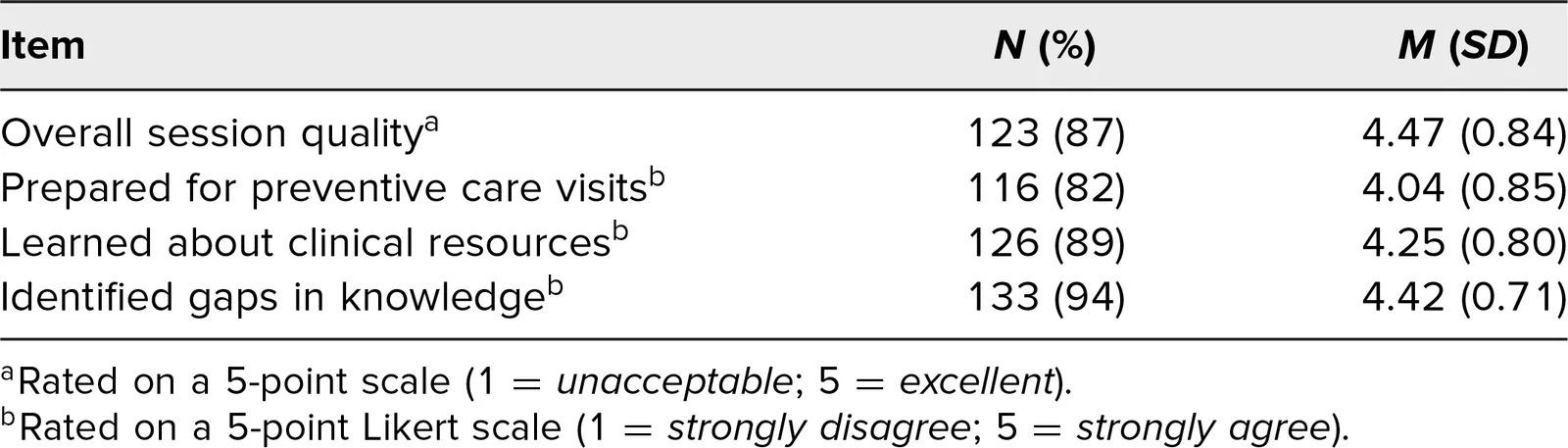
Learner Postsession Reaction Outcomes (*N* = 142)

Open-ended comments reinforced these findings and highlighted the value of the format itself. Learners frequently described the session as interactive, engaging, and more effective than traditional lecture formats. Representative comments included: “I really liked the self-directed learning aspect of this session, it could have been a really long boring lecture but making it a game made everyone engaged and focused on learning all of the information” and “This session encouraged active student participation, tested student understanding, and promoted teamwork through its use of fun and interactive puzzles.”

### Self-Reported Takeaways

Open-ended responses provided illustrative examples of what learners felt they gained from the session, spanning the content domains addressed in the activity (Table 2). Developmental milestones and developmental surveillance were the most frequently cited takeaway.

**Table 2.**
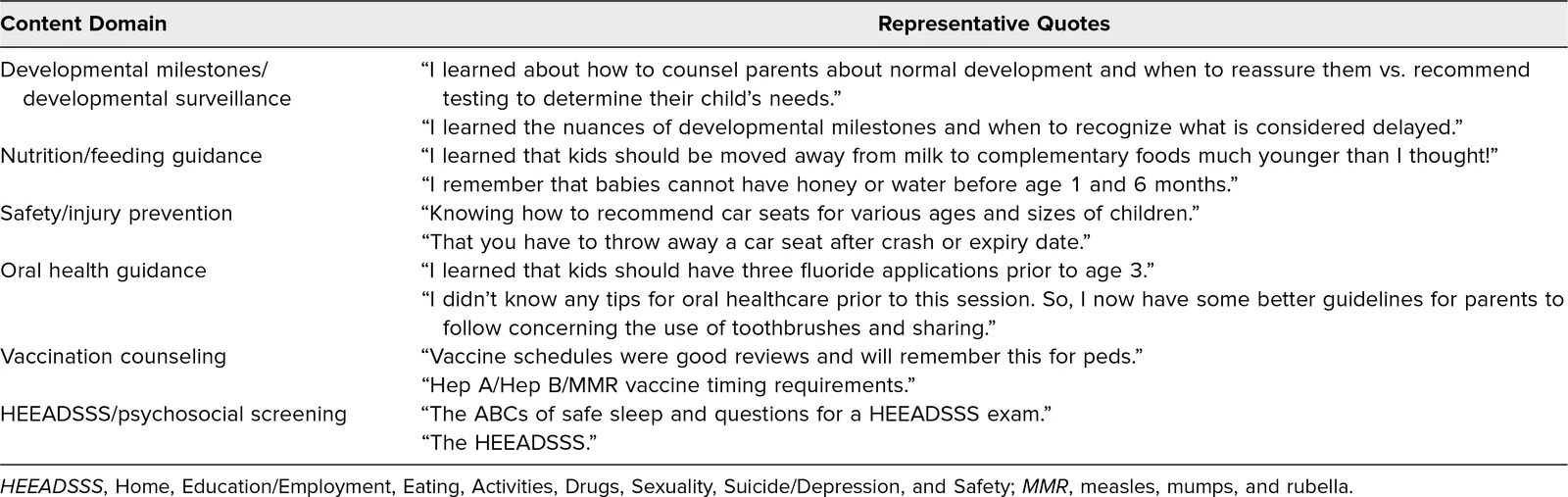
Illustrative Self-Reported Takeaways from Open-Ended Responses

Constructive feedback also identified areas for refinement. Some learners noted that the competitive pace occasionally made it difficult to pause and consolidate learning during the activity. Others identified discrepancies in developmental milestone materials or answer keys, and suggested smaller groups or more explicit postsession review. These comments informed subsequent revisions to the activity materials and supported the importance of a structured debrief to reinforce key concepts and clarify areas of confusion.

### Faculty Feedback

Faculty facilitators also reported high learner engagement and enjoyed teaching the session, describing it as energetic, highly engaging, and more participatory than traditional formats. Representative quotes include: “Creating a game-like feel with incentive for completing it as fast as possible really made the session fun and engaging for everyone” and “The escape room format is extremely engaging, and the students rave about it afterwards. Anecdotally, students tell me that it is their favorite session because it is quick-paced and fun—and they learn a lot from it. From a teaching perspective, it is enjoyable to teach (in part because the students are so engaged).”

### Pilot with Medicine/Pediatric Residents

The pilot with the 8 medicine/pediatrics residents, conducted before data collection with our second student cohort, also yielded favorable comments, with learners describing the activity as a high-quality, interactive way to review developmental content. Representative comments include: “Enjoyed the interactive nature of the presentation!” and “Great review of developmental peds.”

## Discussion

This activity addressed a common challenge in pediatric education: teaching the breadth of health supervision in a way that is clinically relevant and memorable. In a distributed clerkship model, where exposure to well-child care varies by site, the escape room created a shared, structured experience emphasizing application over passive review.

Learner response was strongly positive. At Kirkpatrick Level 1, most students rated the session highly and described it as engaging, interactive, and preferable to a traditional lecture format, consistent with prior literature on escape rooms and game-based learning, improving engagement, motivation, and clinical application.^16-24^

Learners’ self-reported takeaways (Table 2) spanned the content domains addressed in the session and closely matched the session's learning objectives, suggesting the activity reinforced clinically meaningful content in a way that was memorable for students. While we do not interpret these self-reported responses as an objective measure of learning (ie, Kirkpatrick Level 2), these comments are valuable as they reflect students’ perceptions of what they learned rather than whether they liked the session.

A major strength of this curriculum was requiring learners to use the same clinical resources they would use in practice; students located, interpreted, and applied tools they could later revisit in the clinic. This reflects real practice, where effective care depends on using trusted resources efficiently and on foundational knowledge—a practical emphasis that may be especially valuable for students in a distributed clerkship model.

The format also aligns with established principles of adult learning theory. Learners moved through the session with a high degree of autonomy, identified gaps in their understanding, and received immediate feedback and an opportunity for reflection.^24,25^ The activity reinforced prior knowledge, introduced new content, and gave learners guided practice using preventive care resources.

Learner comments also identified areas for improvement. While the competitive structure increased energy and engagement, some learners reported feeling rushed, and others identified inconsistencies in developmental milestone materials and answer keys. In response, we revised answer keys, strengthened developmental milestone resources, preserved a facilitator-led debrief, and created a structured debrief guide for facilitators (Appendix E) to use in future sessions, so that key teaching points are not lost in the pace of the game.

Limitations include reliance on self-reported reaction and self-reported takeaways, without an objective knowledge assessment, comparison group, or follow-up measure of knowledge retention or clinical application. We chose not to include a formal pre/post knowledge test in this initial evaluation because our primary aim was to assess feasibility, learner reaction, and perceived value of a game-based curriculum before building toward outcome measurement. We agree that this is an important next step. Open-text responses were optional and not completed by all students; many comments were very brief. Because students worked in teams of 3 to 6, successful completion of a room's puzzles reflects team- rather than individual-level achievement of the learning objectives, and some students may not have individually demonstrated each objective as well as team members with more prior clinical experience or whose confidence may have contributed more than others. We also did not track individual completion of the optional preparatory work, so we cannot compare outcomes by prework completion status. Although facilitators reserved time for a debrief and reviewed materials in advance, the debrief was not standardized across sessions, so its depth may have varied by facilitator. We have since developed a structured debrief guide (Appendix E) and added standard facilitator hint guidance (Appendix D). Finally, the curriculum was implemented within a single institution's clerkship structure, which may limit generalizability, although the medicine/pediatrics resident pilot suggests the session may be relevant to a broader group of learners.

This activity was designed to fit within a single 2-hour session inside an existing weekly didactic block in our distributed clerkship curriculum, rather than adding contact time. Running the session required adequate shared space for several concurrent small-group stations and the standardized toolkit in Appendices B and C, with no specialized equipment. Programs with a different curricular structure or without prior foundational instruction could substitute our preparatory materials with additional facilitator orientation as needed. The core station-based, small-group format with a debrief should be adaptable to programs of different sizes, though the content of our puzzles was tailored to our own curriculum and would need review by adopting sites.

Despite these limitations, this escape room offers a practical, low-technology, and adaptable approach to teaching pediatric health supervision. Future work should incorporate an objective knowledge assessment (eg, a brief pre/post quiz aligned with the learning objectives) to evaluate Kirkpatrick Level 2 outcomes, track individual and team performance, formally evaluate feasibility across additional sites, and pilot the structured debrief content in Appendix E to assess whether standardizing this component improves consolidation of learning beyond what was observed with facilitator-led debrief. The format could also be adapted for hybrid or virtual delivery and for learners at different stages of training.

## Appendices


Breastfeeding Slides.pptxCoordinator Assembly Directions.docxEscape Room Guidelines Slides and Toolkit.pptxEscape Room Content and Answer Key.pptxFacilitator Structured Debrief Guide.docxStudent Feedback Questions.docx
*All appendices are peer reviewed as integral parts of the Original Publication.*
