# Supplementary material for: Codes, Clues, and Checkups: Teaching Pediatric Health Supervision to Medical Students with an Escape Room
Source: MedEdPORTAL. 2026 Nov 13;22:11653. doi: 10.15766/mep_2374-8265.11653 (PMC13760381; doi:10.15766/mep_2374-8265.11653)
Supplement: Supplementary file 1 [file mep_2374-8265.11653-s001.zip › B. Coordinator Assembly Directions.docx]

**Appendix B: Pediatric Health Supervision Escape Room**

**Coordinator Instructions**

***COORDINATOR***

***Prep* for the session:**

Students should be reminded to complete Health Supervision pre-work prior to the session

Students should be divided into groups of 3-6 within the same space (see photos)

Coordinator should make sure they have the proper supplies for this activity:

1. Display and hook ups for laptop at the front of the room with facilitator guide pulled up
2. Answer Keys for all 5 ”rooms” at the front of the room for the facilitator
3. Extra pens for each group
4. A timer
5. The Facilitator Guide PowerPoint slides loaded on the computer to project at the beginning
6. A printout of the Facilitator Instructions and Facilitator Guide PowerPoint slides (printed with notes viewable)
7. Students should have access to Student Guide for viewing during session and Session Materials for Take-Home Resources
8. The Facilitator Guide PowerPoint slides loaded on the computer to project at the beginning
9. A printout of the Facilitator Instructions and Facilitator Guide PowerPoint slides (printed with notes viewable)
10. Students should have access to Student Guide for viewing during session and Session Materials for Take-Home Resources
11. A completion certificate for each student
12. Leaderboard spreadsheet for tracking of times
13. 4-6 copies of each of the below folders

- Baby folder which includes:
  - Developmental Milestones Connections
  - Vaccine Crossword
  - Injury Prevention Safe Sleep image
  - Breastfeeding Quiz
- Toddler folder which includes:
- Toddler Trivia Grab Bag
- Developmental Milestones Matching
- Cavity Free at 3 advice
- Kindergarten folder which includes:
  - Car Seat Shopping Spree
  - Nutrition notes
- 4^th^ Grade folder which includes:
  - Puberty Stages Matching Game
  - Firearm Safety Crossword Puzzle
- 8^th^ Grade folder which includes:
  - Adolescent Strands
  - Confidentiality/Consent Sorting
  - Anticipatory Guidance Word Search
  - Mental Health Jumble

*All Images below are Author Owned

Example of a room with 4 groups
