# Supplementary material for: Codes, Clues, and Checkups: Teaching Pediatric Health Supervision to Medical Students with an Escape Room
Source: MedEdPORTAL. 2026 Nov 13;22:11653. doi: 10.15766/mep_2374-8265.11653 (PMC13760381; doi:10.15766/mep_2374-8265.11653)
Supplement: Supplementary file 1 [file mep_2374-8265.11653-s001.zip › E. Facilitator Structured Debrief Guide.docx]

Appendix E: Structured Debrief Guide

# Purpose

The escape room runs fast and is competitive by design, and that's part of what students like about it, but it also means there is not much built-in time to stop and think. Some student feedback suggested the pace made it hard to pause and consolidate what they were learning. The debrief is meant to fix that. Facilitators should spend the last 15-20 minutes of the session walking back through each room with the group, keeping in mind that there is some overlap between rooms, specifically the baby and toddler rooms. Facilitators should use a simple three-step pattern:

- **Describe** what happened. Teams say what the puzzle asked and where they got stuck.
- **Analyze** it. The facilitator clears up any confusion and reinforces the actual clinical concept, using the same review slides already in Appendix D.
- **Apply** it. Students say how they would use this with a real patient or family.

# Timing

Fifteen to twenty minutes at the end, after a winner is declared. While students might still be physically separated into their teams within the room, everyone should be reconvened together paying attention to the facilitator.

Facilitators don't need to read every slide word for word as indicated in slide 85 in Appendix D. What this guide adds is a consistent set of prompts around that same content, so the debrief is consistent across each session and cohort, even when only a few slides get used.

# Room-by-Room Prompt Examples

|  | Slide Content | Describe | Analyze | Apply |
| --- | --- | --- | --- | --- |
| Baby Room | Surveillance vs. screening, CDC milestones, ASQ/SWYC/PEDS (86,87); vaccine schedule and hesitancy (88,89), safe sleep (90)milestones 2-9 months (91-94), nutrition 0-12 months (95,96) | "What milestone or vaccine question tripped your team up?" | Clarify surveillance vs. screening; walk through the presumptive/motivational-interviewing language on slide 89. And review the safe-sleep checklist slide 90 | "A parent says they're worried the vaccine will make their baby sick. What do you say first?" |
| Toddler Room | Milestones 12-24 months (97-99); nutrition 12-24 months (100) | "What surprised you about the nutrition recommendations?” | Discuss the transition to whole milk around age 1 and guidelines for toddler eating as well as importance of family meals | "A family is formula feeding and wondering what the best time and method is to switch to whole cows’ milk. How would you counsel them?” |
| Kindergarten Room | Milestones 3-4 years old (101,102), car seat safety (103,104), and nutrition ages 2-5 (105) | “Car seats can be confusing-what was a key takeaway for counseling you that you took from the game?” | Go over the types of car seats and general guidelines for advancing (infant rear- facing, rear- facing toddler, forward-facing toddler, booster seat). Review picky eating guidance. | “You are seeing a patient for her 2.5 year WCC- she is at the 3^rd^ percentile for height and parents want to know what car seat she should be in- how do you advise them?” |
| Fourth-Grade Room | Firearm-related injury and safe storage (106) | “How many of you have actually heard this asked during a well child visit with your preceptor?- did you miss any of the anticipatory guidance words in the puzzle?” | Go over the guidelines and how to appropriately ask and counsel families about firearm safety. | “Your patient tells you they have a gun at home that is unloaded but not locked up- but the 9 year old knows all about gun safety- what are your next steps in counseling?” |
| Eighth-Grade Room | HEEADSSS domains, confidential visit, depression/substance screening, and confidential services (107) | "What did your team name as the HEEADSSS domains — what did you miss?" | Walk through the full HEEADSSS framework and your state's confidentiality rules. | "How do you open a confidential conversation with a 14-year-old when a parent is in the room?” |

# Closing

End by asking each team for one thing, from any room, they would do differently at a real well-child visit tomorrow. It's a quick way to send students out with something concrete instead of just the memory of a fun activity, and it's the piece students and faculty both flagged as missing when things ran long.

# A Note on Pace

Students are under time pressure and a bit of competitive adrenaline for most of the session, which may not be the best state for actually absorbing new material. This is why the debrief is so important. Facilitators shouldn't skip it if a session runs late. Completing some of the debrief is better than skipping it altogether although facilitators should try to be aware of timing and leave appropriate time at the end to complete the full debrief.
