# Supplementary material for: Codes, Clues, and Checkups: Teaching Pediatric Health Supervision to Medical Students with an Escape Room
Source: MedEdPORTAL. 2026 Nov 13;22:11653. doi: 10.15766/mep_2374-8265.11653 (PMC13760381; doi:10.15766/mep_2374-8265.11653)
Supplement: Supplementary file 1 [file mep_2374-8265.11653-s001.zip › F. Student Feedback Questions.docx]

Appendix F: Student Evaluation Questions

- Time was given at the end of the session to complete these online questions and emailed out after the session for completion.

Questions 1-2: Answer choices on a Likert Scale (Excellent, Very Good, Acceptable, Borderline, Unacceptable).

1. What was the overall quality of this session content?
2. What was the overall quality of the teaching provided by the instructor?

Questions 3-9: Answer choices on a Likert Scale (Strongly Agree, Agree, Neutral, Disagree, Strongly Disagree).

3. I would recommend including the session in the curriculum for the LIC next year.
4. Overall, I enjoyed the escape room activity.
5. The escape room was useful for my learning.
6. Playing the escape room made me want to learn more about Pediatric Health Supervision.
7. The escape room activity helped me learn about clinical resources important to Pediatric Supervision.
8. The escape room activity helped me identify gaps in my knowledge.
9. The escape room activity helped me feel more prepared for seeing children for preventive care.

Questions 10: Open Ended Response

10. State at least one item you learned that will apply to patient care.
